# Field Evaluation of Integrated Management for Mitigating Citrus Huanglongbing in Florida

**DOI:** 10.3389/fpls.2018.01890

**Published:** 2019-01-31

**Authors:** Muqing Zhang, Chuanyu Yang, Charles A. Powell, Pasco B. Avery, Jihua Wang, Youzong Huang, Yongping Duan

**Affiliations:** ^1^Agricultural Science College, Guangxi University, Nanning, China; ^2^Indian River Research and Education Center, University of Florida, Fort Pierce, FL, United States; ^3^US Horticultural Research Laboratory, USDA-ARS, Fort Pierce, FL, United States

**Keywords:** citrus huanglongbing, integrated management, chemotherapy, thermotherapy, *Candidatus*, Liberibacter asiaticus, Las bacterial titers, HLB index

## Abstract

Citrus huanglongbing (HLB) is extremely difficult to control because the psyllid-transmitted bacterial pathogen resides inside the citrus phloem and the disease is systemic. In Florida, the nine billion dollar citrus industry has been significantly impacted by severe HLB epidemics. To combat citrus HLB, in this study we implemented an integrated strategy that includes chemotherapy, thermotherapy, and additional nutrition treatment in three different field trials over three consecutive years. In these trials, only trees already showing HLB symptoms with Ct values ranging from 25.1 to 27.7 were selected for treatments. To assess the complex interactions, we used several methods for evaluating the effectiveness of integrated management, including the slopes (b) of the Ct increase (dy/dt), the pathogenic index (PI) and the decline index (DI) from Ct value and tree scores, and the therapeutic efficacies from PI and DI. This comprehensive analysis showed that most of the tested chemicals were effective to some degree in killing or suppressing the Las bacterium, with higher therapeutic efficacies seen for Grove B, where citrus trees were severely affected by HLB, and it had a higher number of psyllids, relative to Grove E and P in the first 2 years. Trunk-injected penicillin G potassium was the most effective chemical treatment in all groves, followed by Oxytetracycline Calcium Complex, and Silver Nitrate delivered as foliar sprays. Although the steam heat treatment and additional nutrition did not eliminate or suppress Las over the long term, these treatments did positively affect tree growth and recovery in the short term. Overall, our results provide new insights into HLB control method and strategy for integrated management for HLB epidemic plantations.

## Introduction

Citrus huanglongbing (HLB) is currently the most devastating citrus disease worldwide. In the state of Florida, millions of infected plants have entered a severe stage of decline with premature fruit drop and dieback. HLB in the United States is associated with *Candidatus* Liberibacter asiaticus (Las), which is transmitted by the Asian citrus psyllid *Diaphorina citri*. Since first documentation of citrus HLB in Florida in 2005, citrus production had declined from 170 million boxes to fewer than 80 million boxes in 2015–2016 (Neupane et al., 2016) due to HLB in addition to adverse environmental conditions. The production in 2017–2018 was an even smaller harvest of 45 million boxes (https://www.nass.usda.gov/Publications/Todays_Reports/reports/crop0518.pdf).

Furthermore, citrus producers in Florida face a different situation from those in China and Brazil, where relocating citrus to new areas is an effective strategy to establish new plantings of HLB-free citrus. Florida citrus growers have no choice other than to produce citrus on their currently available acreage, regardless of the presence of HLB. However, the impact of HLB on the Florida citrus industry might be mitigated in at least two ways: (1) reducing the HLB pathogen titers and disease index in currently infected trees in existing groves; and (2) preventing and/or curing new infections in new plantings. Thus, management strategies to kill or suppress the HLB bacterium and restore plant vigor are critical. Based on the complex nature of HLB pathosystem and the shortfalls associated with currently available measures in the field, we implemented an integrated therapeutic approach to mitigate HLB effects by reducing Las titers and disease index in field trial.

This integrated therapeutic approach was developed based on previous accomplishments for HLB control that includes development of a chemical screening system to identify effective chemicals for HLB control (Zhang et al., 2010, 2012). More than 120 chemical compounds were evaluated using the optimized graft-based assay to determine activity against Las bacterium and phytotoxicity (Zhang et al., 2010, 2011, 2012, 2014). Of these, 11 have been advanced into these field trials. Because of the systemic nature of HLB infection and the residency of Las in the phloem of infected trees, and the cuticle wax of citrus leaves, oil-in-water and water-in-oil nano-emulsion for application to the bark and as foliar sprays were developed to increase delivery efficiency, respectively (Yang et al., 2015, 2016a).

In addition to chemotherapy, we demonstrated that heat treatment could eliminate Las under controlled conditions (40–42°C for a minimum of 48 h) (Hoffman et al., 2013). Field trials of this approach showed partial success in portable greenhouses that rely on solar energy (Doud et al., 2017). Problems associated with the implementation of thermotherapy in the field include the need to develop techniques that allow the desired temperatures to be attained and maintained within a designated timeframe in a large-scale setting, as well as the need for methods that allow simultaneous treatment of the roots and canopy of infected trees. In previous study, combination therapies that use both heat and chemicals are effective for mitigating HLB infections in greenhouse (Yang et al., 2016b). Here we described the results of a 3-year field trial conducted in the Indian River to examine the therapeutic efficacy of approaches to mitigate HLB that involved an integrated strategy of chemical treatments coupled with heat treatment and additional nutrition.

## Materials and Methods

### Field Trial

A 3-year field trial was carried out on a randomized split-plot design to combine chemical, heat and additional nutrition treatments in an overlapping experiment. The 12 chemical treatments were delivered as the major block in each location (grove), whereas two heat treatments was delivered in a sub-split plot, and two additional nutrition was given to a sub-sub-split plot. The data were analyzed as a generalized linear mixed model using the SAS procedure GLIMMIX 8.1. The whole block and split plot factors were treated as fixed effects, and the replication and its interaction with the whole-plot factor were treated as random effects. Differences among treatments were determined with the LINES option of LSMEANS.

These treatments were applied to 192 HLB-positive red grapefruit/sour orange rootstock trees in each of three commercial groves [Groves B (high HLB infection rate), E (moderate HLB infection rate) and P (moderate HLB infection rate)] located in the Indian River. The trees were aged between 3 and 4 years, and the heights ranged from 4 to 8 feet. Additional nutritional and heat treatments were applied to 96 trees in each grove, respectively.

### Chemotherapy

Twelve chemical treatments (Table 1) were applied to 16 tree replicates in each grove. All chemicals were applied via foliar spray. In our previous study, trunk application of penicillin was high efficacy against Las (Yang et al., 2016b). Therefore, in this paper, penicillin was given via trunk injection as a positive control.

**Table 1:** Type and concentration of chemicals applied to citrus trees in the field.

**Chemicals**	**Code**	**Concentration and application additives**
Mycoshield® Oxytetracycline Calcium Complex	OXY	200 ppm with Tactic® surfactant
Firewall® Streptomycin Sulfate	Strep	11 ounces per acre with Tactic® surfactant
Aliette® WDG Aluminum tris (O-ethyl phosphonate)	ALI	Five pounds per acre with Kinetic® surfactant
Silver Phosphite	SP	Two quarts per acre and a proprietary SP trunk drench formulation applied at 1 liter per tree with Kinetic® surfactant
Silver Nitrate	SN	200 ppm concentration with nano-cre emulsion
Carvacrol	CARV	500 ppm with Makon 12 emulsion
P-Cymene	PCY	500 ppm with Makon 12 emulsion
Validoxyamine	VA	200 ppm with nano-cre emulsion
Zhongshengmycin	ZS	200 ppm with nano-cre emulsion
Sulfamethoxine sodium	SDX	100 ppm with nano-cre emulsion
Penicillin G potassium (Positive control)	PEN	500 ml 5,000 ppm solution, applied by trunk injection 3 times at approximately 1 week intervals
Untreated control	CK

### Thermotherapy

Heat treatment was given using mobile steam equipment to inject the steam under a portable tent on June 18th, 2015 (grove B) and June 22nd, 2015 (grove E and P). The temperature of the air around the tented tree was raised to 51.6–53.3°C for a period of 30 s. Temperatures were monitored with a sensor placed in each tree.

### Additional Nutrition

In the three groves, Renew® 3-18-20 (Plant Food Systems, Inc., Zellwood, FL) and Citrus Mix (Miller Chemical & Fertilizer, LLC, Hanover, PA) were delivered as foliar sprays at rates of 1 gallon (1 gallon = 3.78541 L) and 1 pound (1 pound = 0.45359 kg) per acre (1 acre = 0.40469 hectare), respectively. Although the chelated micronutrient mix (Citrus Mix) is generally not phytotoxic on its own, its application in combination with Renew® resulted in excessive leaf drop. Therefore, Citrus Mix was replaced by R-TRx liquid micronutrient mix (Diamond R Fertilizer Company, Ft Pierce, FL) and applied at 1 gallon per acre beginning with the second round of treatments, and Renew® 3-18-20 also was applied at 1 gallon. No leaf drop was observed following application of this treatment. In order to provide sufficient nutrition to HLB-affected citrus, the nutritional therapy treatments were applied in addition to the grower/cooperator fertilizer programs, which generally followed UF/IFAS recommendations. Trial Grove B received 3 soil applications of a complete, dry fertilizer mix each year. Trial Grove E received 2 soil applications of a complete slow-release dry mix and monthly applications of N, P, K and micronutrients through the microsprinkler irrigation system each year. Grove P received three soil applications of a complete, dry soluble fertilizer mix and monthly N, P, K, and micronutrients through the microsprinkler irrigation system each year.

The first foliar nutritional treatment application contained Renew® 3-18-20 (urea, dipotassium polyphosphate, phosphorus acid, salicylic acid) at a 1 gallon per acre rate and 1 pound Science Citrus Mix EDTA-chelated micronutrient mix, combined in a tank mix and applied as a foliar spray. Despite of the chelate being an excellent product, when combined with the Renew product, it caused excessive leaf drop. Subsequent treatments throughout the trial contained 1 gallon per acre of RTRx liquid micronutrient mix (zinc, manganese, copper, iron, boron, molybdenum, sulfur and biostimulants as a glucoheptonate complex) instead of the Science Citrus Mix EDTA chelate. The new nutritional tank mix used in the subsequent treatments did not cause any leaf drop.

All chemical treatments were applied in addition to the grower/cooperator spray programs, which did include regular pesticide for psyllid control and foliar micronutrient products, but excluded any antimicrobial treatments. Foliar spray treatments were applied each growing season during the spring (three sprays), summer (one spray) and fall (three sprays) growth flushes (total of seven sprays per year). The first application was applied when the new growth flush first appeared and subsequent applications were made at 14 day intervals so that all new growth would be quickly covered with at least one application. Foliar applications were made with a 100 gallon tank sprayer equipped with a 50 psi (1 psi = 0.06895 bar) handgun calibrated to deliver approximately 150 gallons per acre. Each tree received about 1.0 gallons of finished spray that provided thorough coverage for each tree selected for the trial. Spray treatments were mixed in 25 gallon batches, except for the nutritional treatments that were mixed in 100 gallon batches to spray 96 trees. To avoid residual material on harvested fruit, the last spray of chemical and nutritional treatments delivered by foliar application began on September 25 and ended on October 10, 2015.

Penicillin injection treatments were carried out by drilling two injection sites 2 inches (1 inch = 2.54 cm) deep in the trunk of the treated trees and inserting 7/32 inches (= 0.56 cm) injection needles attached by hoses to a plastic bag hung above the injection points, which allowed the solution to enter the tree via gravity feed. New injection sites were drilled in the penicillin (PEN)-treated trees for each time of application (Shin et al., 2016).

### Las Bacterial Quantification and Pathogen Index

Branches showing typical HLB symptoms were tagged for quantification of Las bacterial titers at the beginning of the experiment and were used for sampling throughout the study. Samples of mature leaves (six leaves) from the tagged symptomatic branch were taken at 4-month intervals between April 2015 and December 2017 for a total of nine samples per tree. Samples were kept cool and out of direct sunlight. DNA from these samples was extracted for quantification of the Las bacterial titer. Total 1,728 leaf samples taken from trees in each of the groves were assayed for Las bacterium by qPCR. Cycle threshold (Ct) value was measured by real-time PCR using previously described primer sets and probes (Li et al., 2006). Las bacterial titers were assigned to categories 0 to 4 based on Ct values where: category 0 = Ct ≥36.0; category 1 = 32.0 ≤ Ct <36.0; category 2 = 28.0 ≤ Ct <32.0; category 3 = 24.0 ≤ Ct <28.0 and category 4 = Ct <24.0. The pathogenic index (PI) used to evaluate Las bacterial titer was calculated for each treatment as follows (Yang et al., 2016b):

$$\begin{array}{l} \text{PI}=\sum_{\text{n}=0}^{4}\frac{\text{Sum of all numerical grades}}{\text{Total number of plants counted}\times \text{maximum grade}}\times 100 \end{array}$$

### Tree Scoring and Decline Index

Tree health was evaluated using scores based on the observable amount of HLB decline, also known as decline indexing (DI). The same skilled field manager scored all 192 treated trees at 6 month intervals over 3 years using a 0–4 scale where category 0 = No HLB symptoms, normal fruit load and size, normal leaf size and growth flushes; category 1 = Some HLB symptoms, modest fruit load and normal fruit size, mostly normal growth flushes; category 2 = Some HLB symptoms, modest fruit load with some smaller fruit sizes, most growth normal; category 3 = Obvious HLB symptoms, light fruit load and multiple small fruit sizes, modest or no growth flushes; category 4 = Obvious HLB symptoms, including small leaves and dead wood, small fruit size and virtually no new growth (see Field Trial Evaluation Methods for Growers at https://citrusrdf.org/wp-content/uploads/2012/10/Citrus-Expo-2016-Slinski.pdf).

The DI was calculated for each treatment as follows:

$$\begin{array}{l} \text{DI}=\sum_{\text{n}=0}^{4}\frac{\text{Sum of all numerical scores}}{\text{Total number of plants counted}\times \text{maximum score}}\times 100 \end{array}$$

### Psyllid Monthly Counts

Each month the incidence of psyllids in each citrus grove was determined using the tapping method (Qureshi et al., 2014). In each grove, 40 random trees, either per row or per bed, were sampled with four tap samples taken per tree. For each tap sample, one of four randomly chosen branches was tapped three times using a PVC pipe rod and insects were collected on a single sticky card attached to a clipboard placed below the branch or branches. The number of psyllids that landed on the sticky card was recorded for 16 trees and 10 card samples were taken per grove. Significant differences among the groves were determined by ANOVA and *ad hoc* means were compared using a Tukey's test (α = 0.05).

### Statistical Analysis

To identify differences among the rates of increase in Las bacterial titer, Ct values (Ct) for each treatment and Ct data (y) over time were fitted to linear [t = t/y] models, according to Madden's method (Madden and Ellis, 1988). Rates of Ct increase for the superior model were then compared using a *t*-test to determine significant differences. To examine the dynamics of HLB infection, Ct increase (y/t), the rate of Ct increase (dy/dt), and the acceleration/deceleration of the epidemic [(dy/dt)/dt] were calculated for each of the treatments, wherein y = Ct value for each treatment and t = time in months. Linear regression was used to examine the slopes (b) of the [(dy/dt)/dt] data points, wherein the arithmetic sign of the slope, +b and -b, is indicative of the rate of acceleration and deceleration in the epidemic growth rate, respectively. Slope comparisons were made by *t*-tests, wherein t = (b_1_-b_2_)/(SE_b1_-SE_b2_), and b_1_ and b_2_ are the slopes of the regression lines to be compared. SE_b1_ and SE_b2_ are the respective standard errors of the slopes.

To evaluate the effect of each treatment, both PI and DI were used to calculate the efficacy of each treatment (antimicrobials, heat and additional nutrition). Without considering the background effect of the treatment, the absolute therapeutic efficacy (E_abs_) was simultaneously calculated from the DI or PI for each treatment by:

$$\begin{array}{l} {\text{E}}_{\text{abs}}=\frac{\text{D}{\text{I}}_{\text{ck}}-\text{D}{\text{I}}_{\text{tr}}}{\text{D}{\text{I}}_{\text{ck}}}\times 100 \end{array}$$

Where DI_ck_ = DI of control; DI_tr_ = DI of treatment

The control increment of the DI (Δ_ck_) was calculated by:

$$\begin{array}{l} \Delta_{\text{ck}}=\frac{{\text{DI}}_{0}-{\text{DI}}_{T}}{{\text{DI}}_{0}}\times 100 \end{array}$$

Where DI_0_ = DI of control at pre-treatment; DI_T_ = DI of control at post-treatment.

To avoid background effects of the treatment, the relative therapeutic efficacy (E_r_) for each treatment was determined according to the following equation adopted by Rewal and Jhooty (1985):

$$\begin{array}{l} Er=\frac{DI_{0}\times \left(1+\Delta_{ck}\right)-DI_{t}}{DI_{0}\times \left(1+\Delta_{ck}\right)}\times 100 \end{array}$$

Where DI_0_ = DI at pre-treatment; Δ_ck_ = the control increment of DI; DI_t_ = DI at post treatment.

## Results

### Titers of Las in HLB-Affected Trees Treated by Integrated Managements

**Figure 1:**
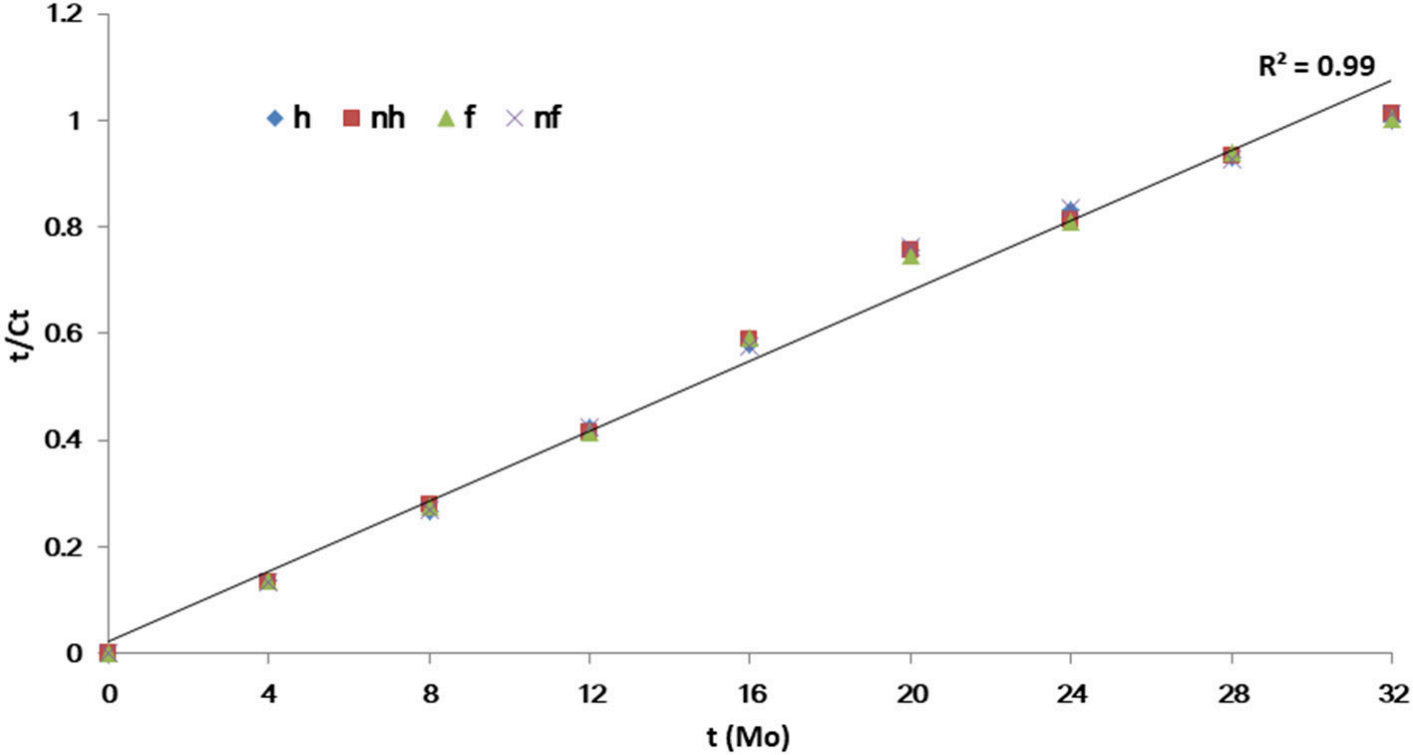
Linear [*t* = t/y] models regression of Ct value (y) over time (t) for heat and additional nutrition treatments.

**Figure 2:**
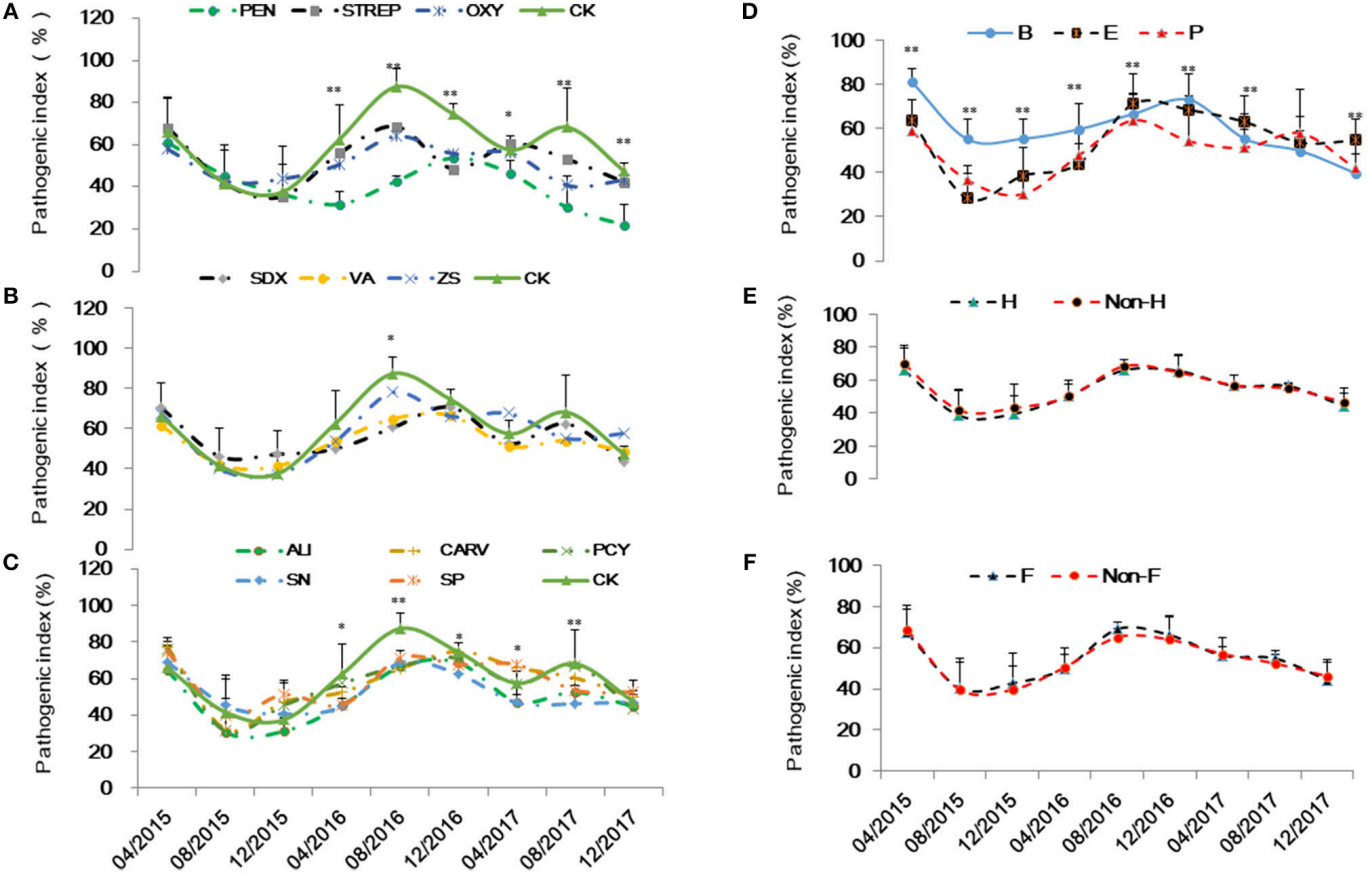
Pathogenic index of the integrated management by chemical (**A** and **B**: the abbreviations listed in Table 1), grove (**C**: groves **B,E** and P), heat (**D**: H, heat treatment; Non-H, without heat treatment) and additional nutrition (**E**: F, additional nutrition; Non-F, without additional nutrition).

Based on visual observation and qPCR quantification, Grove B had the highest HLB infection rate at 95.4%, followed by Groves E and P with rates of 39.4 and 45.2%, respectively. Variance analysis showed no significant differences in Ct values ranged from 25.1 to 27.7 among the HLB-affected trees at same grove prior to treatments. However, Grove B had significantly lower Ct values (i.e., higher Las titers) compared to Groves E and P. Ct values also varied according to seasonal fluctuations and associated integrated treatments. Ct data (y) over time (32 months) were fitted to linear [t = t/y] models for each treatment. The slopes (b) and intercept (a) in this paper indicated that acceleration/deceleration of the epidemic of HLB. Although the *t*-test showed the slopes for the [(dy/dt)/dt] data points significantly differed among all chemical treatments (*p* ≤ 0.001), the intercept of the data points did not (*p* > 0.05). The slope for the positive control PEN treatment was significantly lower than that of the untreated control (CK), followed by OXY, SN, ALI, STREP and VA (Table 2). There were no significant differences in the slopes for values derived for trees subjected to heat treatment (*p* = 0.885) and/or given additional nutrition (*p* = 0.816) (Figure 1). To lessen the background noise of the treated trees, a pathogen index (PI) was calculated for each chemical compound (Figure 2). The PI differed significantly among the groves according to variance analysis (Figure 2C). Relative to Groves E and P, Grove B showed a higher PI in the first year after the initial treatment in April 2015, then decreasing to below that of Grove E in the third year. Although no significant difference in PI was found among the chemical treatments in the first year after initial treatment, the differences among chemical treatments later attained significance. PEN had the highest Ct and the lowest PI, and thus was the most effective at eliminating Las, followed by OXY and SN. Compared to the untreated CK, samples from trees treated with ZS, PCY and SDX showed no significant differences in either Ct value or PI.

**Table 2:** Intercepts (a) and slopes (b) of Ct (y) over time (t) calculated from linear [*t* = t/y] models for each treatment.

**Chemicals**	**Grove B**		**Grove E**		**Grove P**		Slope (b)*
	**a**	**b**	**a**	**b**	**a**	**b**	**Mean ±*Sd***
ALI	0.0150	0.0326	−0.0198	0.0359	0.0019	0.0324	0.0336 ± 0.0020^ab^
CARV	0.0119	0.0347	−0.0157	0.0358	−0.0022	0.0351	0.0352 ± 0.0006^a^
CK	0.0388	0.0327	−0.0023	0.0360	−0.0148	0.0372	0.0353 ± 0.0023^a^
OXY	0.0204	0.0332	0.0064	0.0340	0.0070	0.0307	0.0326 ± 0.0017^ab^
PCY	0.0359	0.0324	−0.0150	0.0365	−0.0068	0.0353	0.0347 ± 0.0021^a^
PEN	0.0291	0.0294	0.0111	0.0304	0.0170	0.0301	0.0300 ± 0.0005^b^
SDX	0.0273	0.0323	−0.0024	0.0343	−0.0147	0.0362	0.0343 ± 0.0020^a^
SN	0.0170	0.0320	−0.0067	0.0351	0.0063	0.0327	0.0333 ± 0.0016^ab^
SP	0.0326	0.0334	−0.0131	0.0363	−0.0054	0.0340	0.0346 ± 0.0015^a^
STREP	0.0236	0.0321	−0.0273	0.0376	0.0137	0.0312	0.0336 ± 0.0035^ab^
VA	0.0292	0.0337	−0.0101	0.0349	−0.0100	0.0333	0.0340 ± 0.0008^ab^
ZS	0.0147	0.0349	−0.0093	0.0367	−0.0143	0.0350	0.0355 ± 0.010^a^

* *Different letter showed significance at 0.05 levels*.

### Tree Scoring and Decline Index (DI) of the HLB-Affected Trees Treated by Integrated Managements

**Figure 3:**
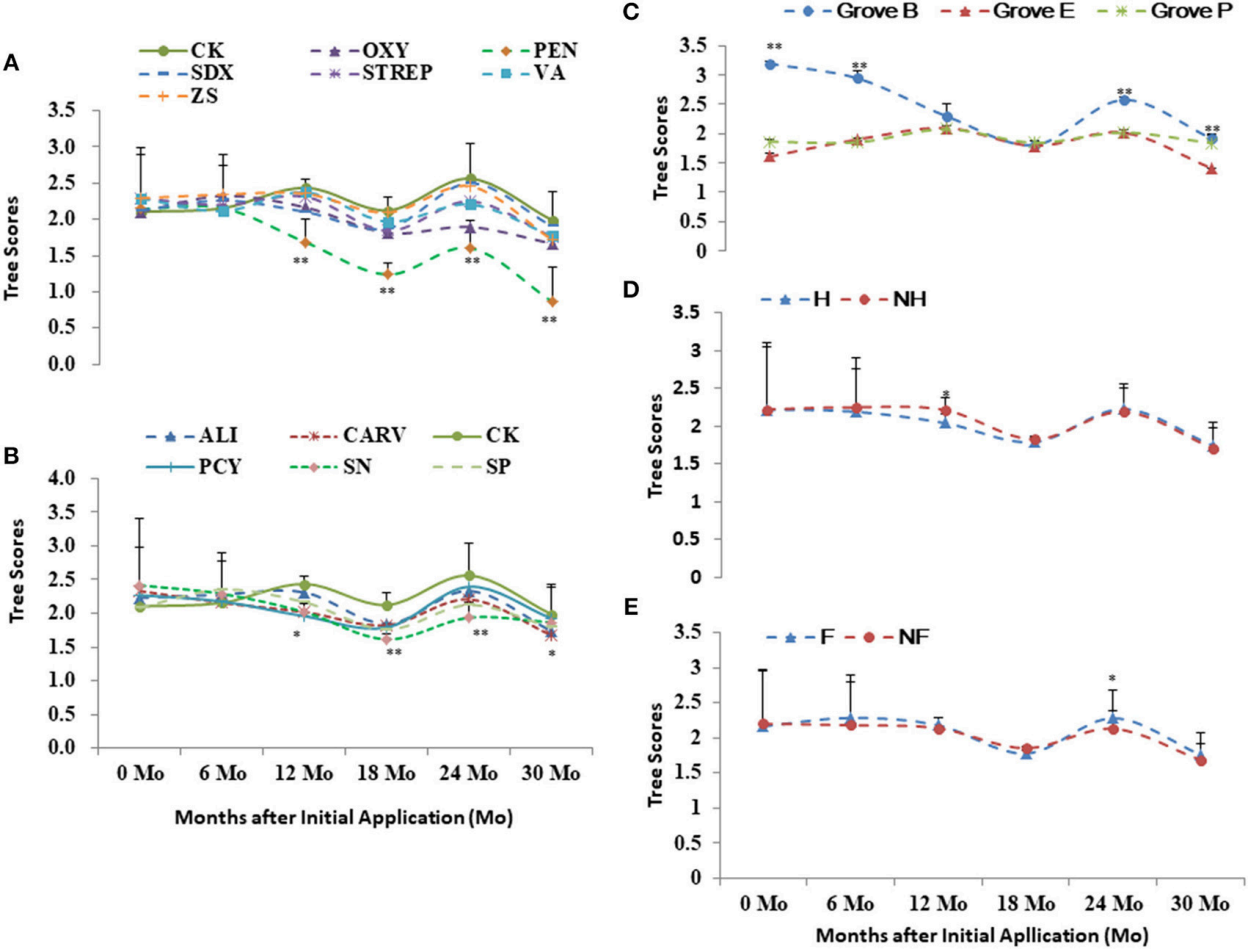
Tree scores of the integrated managements treated by chemical (**A,B**: the abbreviations listed in Table 1), heat (**D**: H, heat treatment; Non-H, without heat treatment) and additional nutrition (**E**: F, additional nutrition; Non-F, without additional nutrition) in each grove (**C**: groves B, E and P). * and ** significance at 0.05 and 0.01 probability level, respectively.

**Figure 4:**
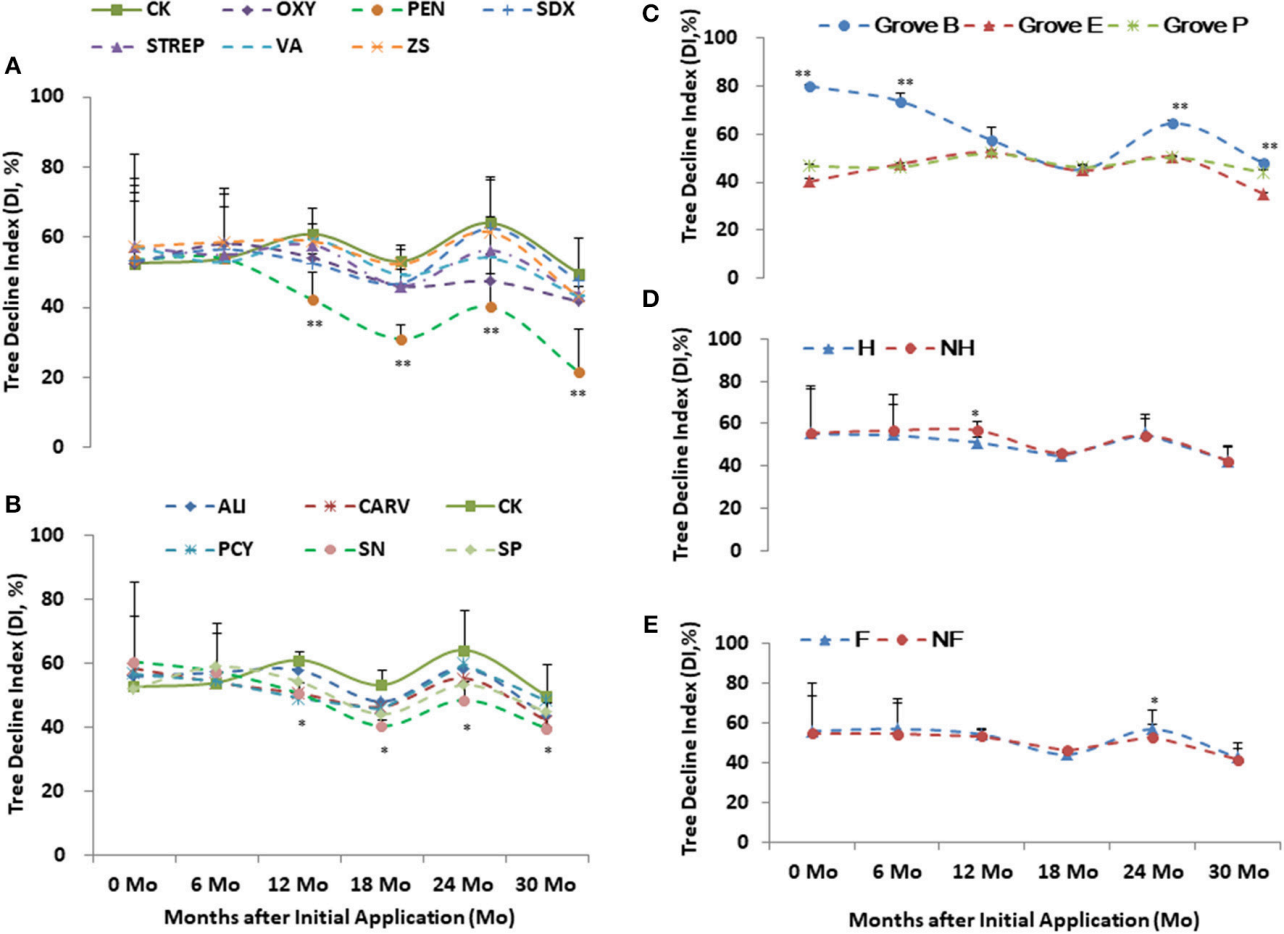
Tree decline index (DI) of the integrated managements treated by chemical (**A** and **B**: the abbreviations listed in Table 1), heat (**D**: H, heat treatment; Non-H, without heat treatment) and additional nutrition (**E**: F, additional nutrition; Non-F, without additional nutrition) in each grove (**C**: groves B, E and P). * and ** significance at 0.05 and 0.01 probability level, respectively.

Grove B had the higher health scores and DI in relative to Grove E and P (Figures 3C, 4C). Higher scores and DI were associated with poorer growth. Differences in the tree scores and DI among the chemical treatments were significant after 1 year post initial application, especially PEN, followed by Oxy and SN. Chemical treatments significantly affected tree health scores 1 year after initial application in all three groves. PEN was the most effective of the chemical compounds, followed by SN and OXY (Figures 2A–C, 3A,B, 4A,B). Compared to without treatments (NF and NH), heat treatment (H) had a short-term positive effect on tree growth and vigor during the first 18 months, whereas additional nutrition (F) had a cumulatively positive effect on growth and vigor over the 2 years after initial application, especially in Grove B (Figures 3D,E, 4D,E).

## Therapeutic Efficacy Of the Chemical Compounds

**Figure 5:**
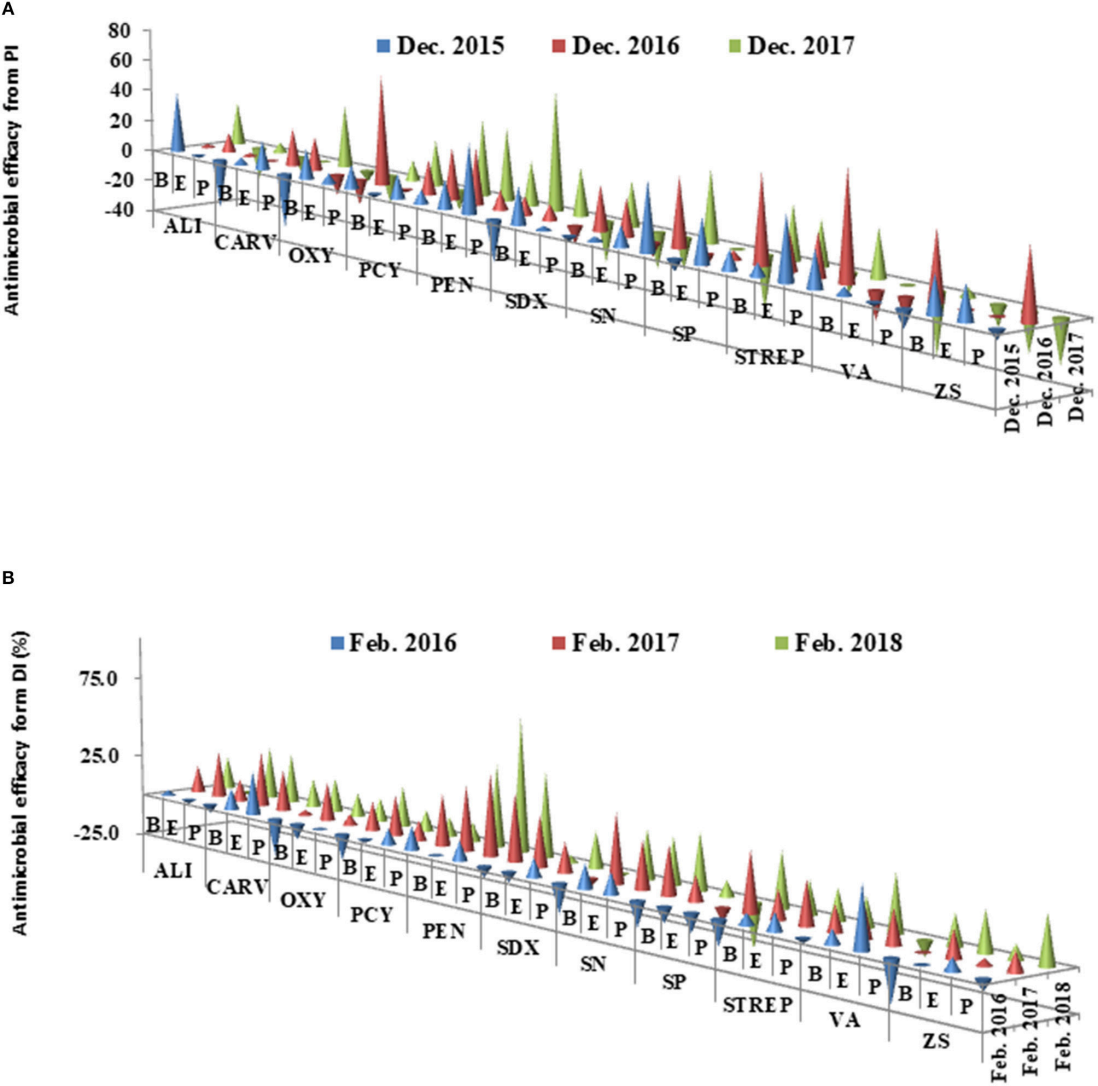
**(A,B)** Antimicrobial efficacies of chemical treatments calculated from pathogenic index (PI) and decline index (DI) of the chemical compounds in three groves from 2015 to 2018. All abbreviations used in this figure were listed in Table 1.

The therapeutic efficacies were calculated from PI and DI for each chemical treatment during the period between 2016 and 2018, respectively (Figure 5). The average therapeutic efficacy was 0.3% in the first year (2016) after the initial application, then increased up to 20% in 2017 (Year 2) and 2018 (Year 3). In Years 2 and 3, all antimicrobial compounds were effective with therapeutic efficacy values that ranged from 4.2% (SDX) to 49.3% (PEN) in Grove B. With the exception of SP in Grove E and SDX in Grove P, the other antimicrobial compounds also effectively promoted citrus growth, with therapeutic efficacies ranging from 5.73% (VA) to 65.2% (PEN). PEN increased its therapeutic efficacy by 43.6% (2017) and 60.3% (2018) for all three groves.

### Psyllid Monthly Variations in Three Citrus Groves

**Figure 6:**
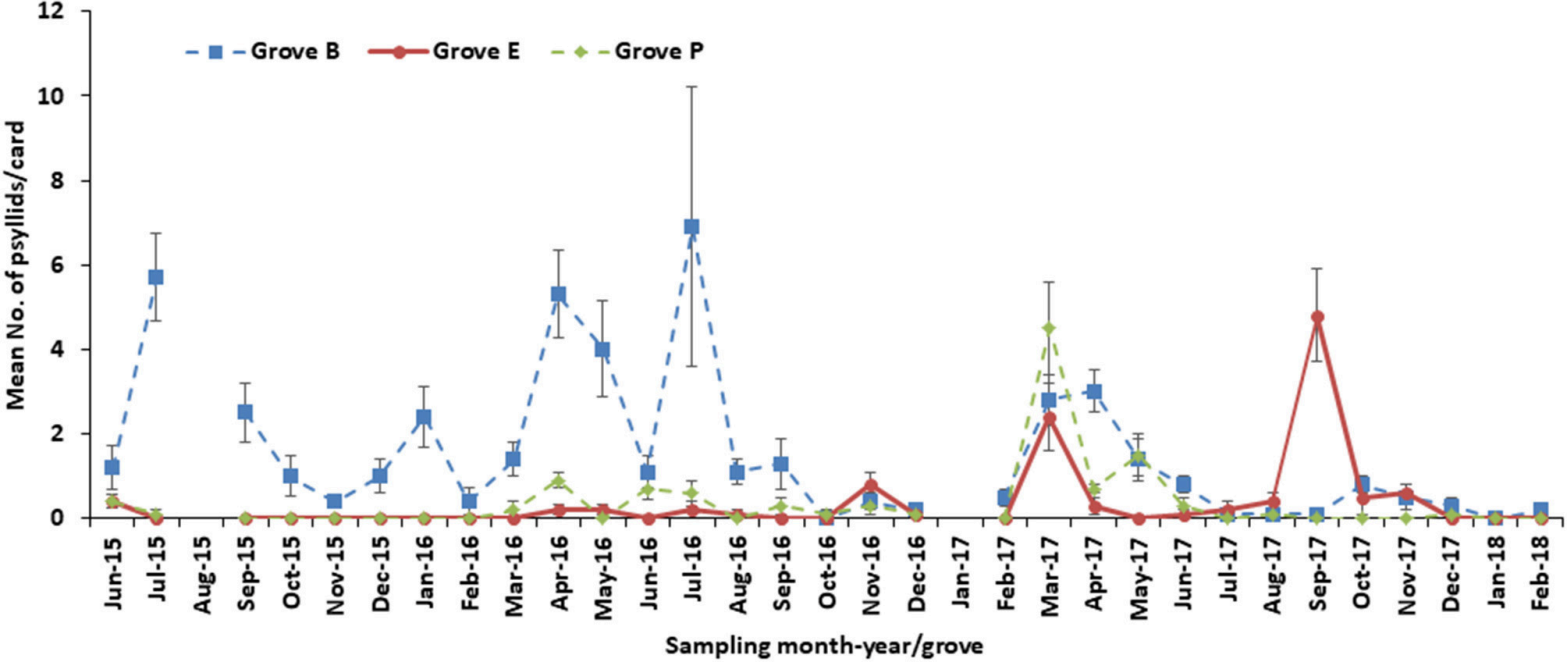
Number of psyllid from tap samples in three groves (B, E and P) from 2015 to 2018.

A similar number of psyllids per card was obtained for all groves during June 2015 (Figure 6), although the actual number of psyllids was higher for Grove B relative to Groves E and P (*F* = 94.19; df = 2, 18; *P* < 0.0001) in July 2015. Between September 2015 and January 2016, psyllids were found only in Grove B. By February 2016, the numbers of psyllids were similar across all three groves, although between March and August 2016 Grove B had more psyllids relative to the other groves. A higher number of psyllids (*F* = 4.38; df = 2, 18; *P* = 0.0282) was seen in Grove B for September 2016, but the psyllid numbers were similar among the three groves between October and December 2016.

In February 2017, after harvest, Grove B had the largest the psyllid population (*F* = 5.69; df = 2, 18; *P* = 0.0122). By March 2017, the citrus tree flowers were blooming, which restricted the spray regime for psyllid control and thus the populations were similar. However, in April 2017 the psyllid population number per tap sample was reduced (*F* = 20.4; df = 2, 18; *P* < 0.0001), with the exception of Grove B, which was similar to the March count. During May 2017, the psyllid population in Groves B and P was similar and higher (*F* = 9.55; df = 2, 18; *P* = 0.0015) than that for grove E. Whereas, the psyllid counts in all groves during June-August 2017 were similar, between September and November 2017, under post-hurricane conditions, the psyllid population significantly differed among the groves. In September 2017 the psyllid mean number was highest (*F* = 35.96; df = 2, 18; *P* < 0.0001) in Grove E. During October 2017, Grove P and Grove E psyllid numbers were similar and lower than that for Grove B (*F* = 5.39; df = 2, 18; *P* = 0.0147). By November 2017, Groves P and B had similar psyllid numbers that were lower than that for Grove E (*F* = 3.94; df = 2, 18; *P* = 0.0381). The mean number of psyllids found per card was similar among the groves during December 2017 and February 2018. No psyllids were found in any grove in January 2018. In nearly all months of the experimental period (June 2015-March 2018) Grove B had a higher mean number of psyllids per card, relative to Grove E and P. Overall, the psyllids were superiorly controlled in Groves E and P compared to that for Grove B.

## Discussion

The citrus industry across the United States relies on finding a sustainable solution that eliminates the spread of HLB and restores HLB-affected trees back to a healthy, productive state through the discovery and application of antimicrobial compounds that either kill or suppress Las. Therefore, our study developed an integrated strategy that includes chemotherapy, thermotherapy and additional nutrition treatment in three different field trials over three consecutive years. To evaluate the complex interaction in the field trial, several analysis methods including the slopes of the dy/dt, the PI and DI from Ct value and tree scores, and the therapeutic efficacies from PI and DI were carried out in this study. Our field plots showed substantial variance in Las bacterial titers (Ct value) that could be due to several factors: (i) an uneven distribution of Las bacterium in the treated trees; and (ii) epidemics occurring in plots subjected to different treatments may peak in terms of HLB incidence or typical symptoms over seasons that may have differing environmental conditions and favorability for citrus HLB (Hu et al., 2017). Taken together, the slope of the Ct value, as well as the PI and DI, reflected the effectiveness of the chemical compounds against Las bacterium in the field. Moreover, the relative therapeutic efficacy is a simple and direct method to evaluate the antimicrobial activity of chemical compounds against Las bacterium that avoids background effects in HLB-affected trees.

In 3-year field trials, several chemical compounds including PEN, OXY calcium complex, silver compounds and Fosetyl-Al, were effective at reducing Las titers in three groves, especially PEN treatment is most effective against Las. Previous reports also showed that PEN is effective in eliminating the Las bacterium and promoting plant growth in graft-inoculated plants (Zhang et al., 2011). PEN is a beta-lactam antibiotic, which binds PEN binding proteins to inhibit cell wall synthesis (Spratt and Cromie, 1988). In addition to its bactericidal effect, PEN can also promote plant growth (Ur Rahman et al., 2004; Zhang et al., 2010).

OXY is short-acting antibiotics that inhibits bacterial growth by inhibiting translation. It passively diffuses through porin channels in the bacterial membrane, binds reversible to the bacterial 30S ribosomal subunit and prevents the aminoacyl tRNA from binding to the A site of the ribosome (Chopra and Roberts, 2001). In the previous studies, OXY can suppress Las titer in greenhouse and field (Schwarz and Van Vuuren, 1971; Aubert and Bové, 1980; Hu et al., 2017). In this study, OXY calcium complex was also effective at reducing Las titers by foliar spray in 3-year field trials. Furthermore, under the emergency Exemption provisions of Federal Insecticide, Fungicide, and Rodenticide Act (FIFRA), Florida has declared an HLB crisis that allows use of OXY calcium complex for controlling citrus HLB by foliar application in Florida.

Silver compounds also exhibit antimicrobial activity toward a range of microbes by altering cell membrane structure and function (Mc Donnell et al., 1999; Pal et al., 2007). As such, silver has been applied to control plant pathogens (Jo et al., 2009; Krishnaraj et al., 2012). These compounds have antimicrobial activity at very low concentrations (<1–10 μmol) that are not toxic to human (Berger et al., 1976), although at higher amounts silver-based compounds can be toxic to mammals as well as freshwater and marine organisms (Bianchini et al., 2002). In this study, silver ions (silver nitrate) showed antimicrobial activity against Las bacterium over 3-year experimental period (Figure 5). Therefore, this chemical compound might be a candidate for controlling citrus HLB pathogens under field conditions.

Fosetyl-Al (ALI) is a phosphonate that demonstrated antimicrobial effects when used to treat infections caused by various microorganisms (Darakis et al., 1997; Panicker and Gangadharan, 1999). Several studies demonstrated that the mode of action of phosphonates can alter fungal metabolism (Guest and Grant, 1991) and cell morphology (Jiang, 1990; Jiang and Grossmann, 1992). ALI suppressed Las titers with PI from 64.6% (April, 2015) down to 44.8% (December, 2017) in 3 years of field trials in this study.

**Figure 7:**
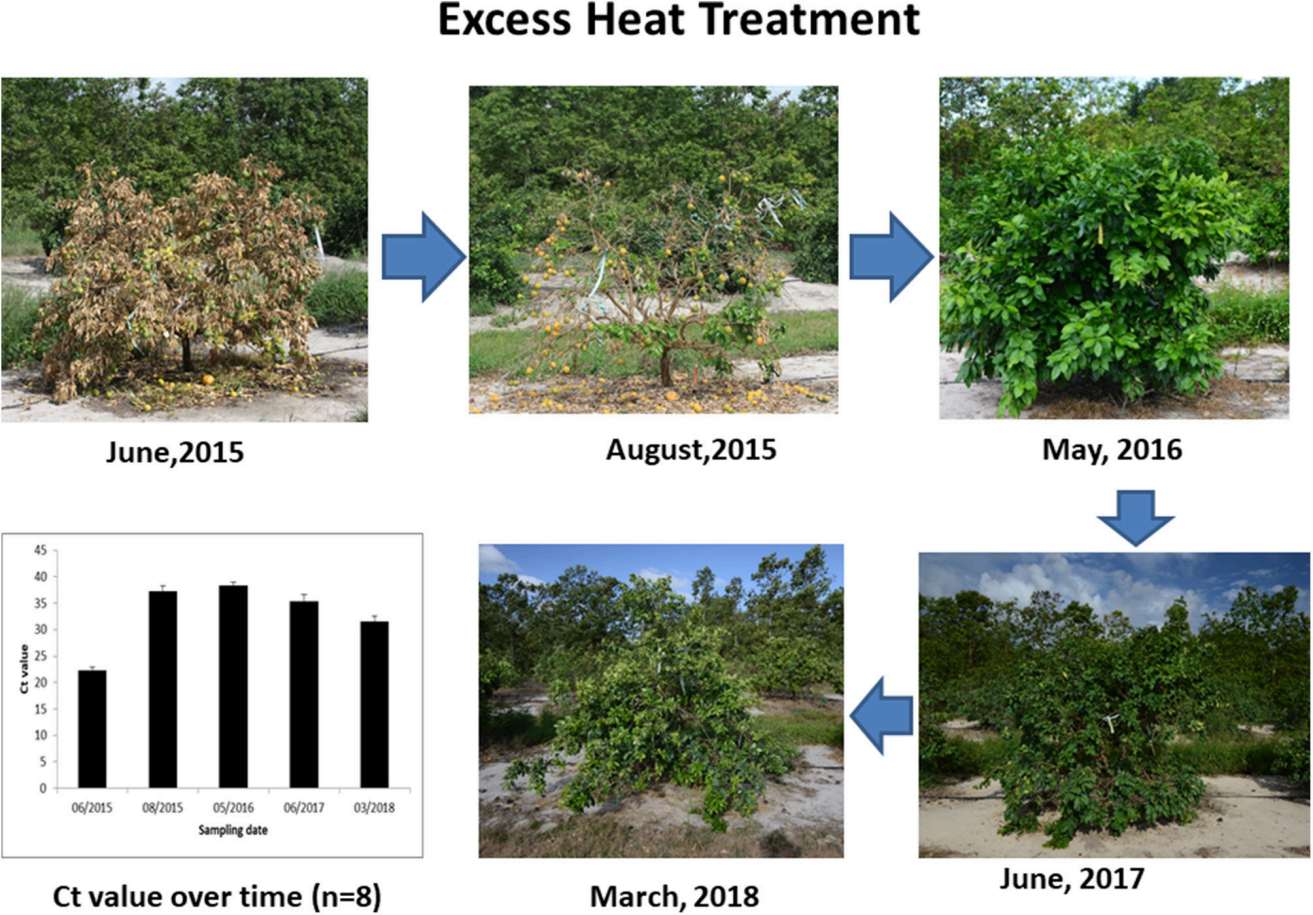
Photos and Ct value of HLB-affected citrus trees before and after over heat treatment.

Both mobile steam heat treatment and additional nutrition did not promote elimination or suppression of the Las bacterium. Mobile steam heat treatment administered as a 30 s exposure to 51.6–53.3°C had a short-term effect on HLB-affected citrus tree growth in the first year after the initial application, whereas additional nutrition had some cumulative effects on citrus tree growth in the third year after the initial application, especially in the severely HLB-affected citrus Grove B. Notably, one HLB-affected tree that did not have Las bacterial infection in the roots was inadvertently subjected to excessive heat treatment that caused loss of all leaves, but this tree exhibited growth after removal of all affected branches as well as branches having a poor appearance (Figure 7). In previous studies, heat treatment enhanced vigor and eliminated Las in HLB-affected citrus (Hoffman et al., 2013; Yang et al., 2016b). Therefore, heat treatment of HLB-affected citrus trees could synergize with chemical compounds.

Foliar micro-nutrient deficiencies are a noted symptom of HLB-affected citrus trees (Spann and Schumann, 2009). Therefore, foliar applications of micro-nutrients have been used by an increasing number of Florida citrus growers to help mitigate HLB-induced deficiencies and counter the debilitating effects of the disease. Our results indicated that nutrition could improve citrus vigor and growth, but was not effective against Las, as previously reported (Stansly et al., 2014). Therefore, nutrition treatment needs to be repeated in control of HLB. However, a combination of heat and nutrition with chemical treatment, especially PEN, SN, and OXY, in an integrated strategy was effective against Las and promoted the growth of HLB-affected citrus trees. This integrated approach may be beneficial for younger planting groves that have low HLB incidence. However, novel, environmentally safe chemical compounds for use against Las will still require screening of effectiveness as part of an integrated strategy to combat the incidence of citrus HLB.

## Author Contributions

CP, YD, and MZ conceived and designed the experiment. CY, PA, JW, and YH performed the experiments. MZ, PA, and CY analyzed the data. CP and YD contributed reagents, materials, analysis tools. MZ, CY, PA, CP, and YD wrote and revised the paper.

### Conflict of Interest Statement

The authors declare that the research was conducted in the absence of any commercial or financial relationships that could be construed as a potential conflict of interest. The handling editor declared a shared affiliation, though no other collaboration with several of the authors MZ, CY, CP, PA, JW, and YH at time of review.
